# An Isolated Intestinal Juvenile Polyp Diagnosed by Abdominal Ultrasonography and Resected by Double-Balloon Endoscopy: A Case Report and Literature Review

**DOI:** 10.3390/diagnostics13030494

**Published:** 2023-01-29

**Authors:** Masumi Nagata, Keisuke Jimbo, Nobuyasu Arai, Kosuke Kashiwagi, Kaori Tokushima, Mitsuyoshi Suzuki, Takahiro Kudo, Toshiaki Shimizu

**Affiliations:** Department of Pediatrics, Faculty of Medicine, Juntendo University, Tokyo 113-8421, Japan

**Keywords:** anemia, double-balloon endoscopy, fecal calprotectin, intestinal juvenile polyp, ultrasonography

## Abstract

Juvenile polyps, typically localized in the rectum and sigmoid colon, are a common cause of pediatric bloody stool. An isolated small intestinal juvenile polyp is uncommon and generally difficult to diagnose. The first case of an isolated juvenile polyp diagnosed by abdominal ultrasonography before acute abdomen had developed and resected by double-balloon endoscopy is presented along with a review of previous reports including this case. A two-year-old Japanese boy was referred to our institute for further evaluation of anemia persisting from one year of age. Laboratory findings showed mild iron deficiency anemia and elevated fecal human hemoglobin (Hb) and fecal calprotectin values. Upper and lower endoscopic findings showed no abnormalities. Because the abdominal ultrasonography performed one year later demonstrated a 15 mm jejunal polyp, combined with a similar finding on small intestinal capsule endoscopy, this was diagnosed as an isolated lesion. The lesion was resected by cautery with double-balloon endoscopy and diagnosed as a juvenile polyp pathologically. All clinical symptoms disappeared, and all laboratory data improved after treatment, without recurrence for more than one year after the procedure. Abdominal ultrasonographic screening and the fecal calprotectin value led to the diagnosis and non-surgical invasive treatment of an isolated small intestinal juvenile polyp.

A two-year-old Japanese boy was referred to our institute for further evaluation and treatment of protracted iron deficiency anemia. The anemia improved with iron supplements, but relapsed following cessation of the medication. Similar episodes were repeated frequently, and fecal human Hb level was also elevated. The patient had no significant personal or family history. At the first visit to our institute, the patient had no growth retardation and no significant abnormalities in vital signs. During the period of iron administration, no physical abnormalities were observed. Moreover, he had no significant external deformities and normal psychomotor development.

The patient’s initial laboratory tests showed Hb 11.2 g/dL, mean corpuscular volume 80.5 fL, mean corpuscular Hb concentration 26.4%, total protein 6.1 g/dL, albumin 4.1 g/dL, immunoglobulin G 461 mg/dL, and ferritin 19 ng/mL. Fecal examinations showed fecal human Hb 215 ng/mL and fecal calprotectin 134 μg/g. Both upper gastrointestinal endoscopy and colonoscopy performed at two years of age showed normal mucosal findings. At three years of age, the patient was re-evaluated by abdominal ultrasonography (AUS) concurrent with capsule endoscopy (CE). Abdominal ultrasonography showed a movable mass lesion in the mid-abdomen, and color Doppler flow showed a blood flow signal toward the mass, leading to the diagnosis of a pedunculated polyp. The polyp was localized in the upper jejunum, with a diameter of approximately 15 mm, and multiple anechoic cysts in the parenchyma, suggesting a juvenile polyp (Figure 1a,b). A same mass lesion was also identified in the upper jejunum by CE, with no abnormalities in the remaining small intestine (Figure 2a).

One month after CE, oral DBE was performed, and an isolated pedunculated polyp, identified 75 cm anorectally from the pylorus, was resected by cautery with a high-frequency snare (Figure 2b–d). The histopathology of the polyp suggesting a hamartomatous polyp, which was consistent with a juvenile polyp (Figure 3). The polyp was removed by DBE with no complications. The patient was discharged a day after polypectomy, following the disappearance of gastrointestinal symptoms and fecal human Hb and calprotectin elevation. Iron supplements were stopped, and the anemia disappeared. At one-year post-treatment, there were no abnormal laboratory data, and no new polyps were identified on abdominal ultrasonographic screening.

Juvenile polyps tend to be localized to the rectum and sigmoid colon and are also associated with iron deficiency anemia due to protracted bloody stool and intussusception involving polyps as the pathologic advanced lesions [1,2]. The pathology is applicable to the small intestine, as well as the colon, particularly in Peutz–Jeghers syndrome, suggesting that small intestinal polyps larger than 15 mm in diameter should be resected to prevent intussusception [3]. Knowledge regarding the diagnostic difficulty and rate of small intestinal polyps is extremely limited because of the insufficient spread of small intestinal endoscopy [4]. In the present case, it was an isolated jejunal juvenile polyp, and occurrence in the small intestine except in juvenile polyposis syndrome is extremely rare and has been reported infrequently [5].

Therefore, a review of this disorder, including the present case, was conducted, examining clinical features, onset age, sex differences, treatments, and examination procedures. Searching in the PubMed database, seven cases were reported between 1981 and 2022, including the present case [5,6,7,8,9,10]. Intussusception and anemia were present in four and three of the seven cases, respectively. Two of the three cases of anemia had suffered for a long duration until diagnosis. The present case also required two years from identification of anemia to diagnosis, suggesting the diagnostic difficulty of an isolated small intestinal polyp [5,7]. No recurrent and malignant cases were found, and no cases of an isolated juvenile polyp were diagnosed as polyposis later. All isolated small intestinal polyps were located in the jejunum, and the diagnosis was established by ultrasonographic identification of jejunojejunal intussusception in three of seven cases, and surgical resection of the polyps was required in five of seven cases. Except for the present case, only one patient with chronic iron deficiency anemia diagnosed by small intestinal CE and who underwent polypectomy using single-balloon endoscopy was not treated surgically [5]. The findings suggest that isolated small intestinal juvenile polyps were diagnosed mainly with the onset of intestinal intussusception, leading to surgery in most patients.

The facts seem to suggest the diagnostic difficulty of this disorder preceding intussusception. Abdominal ultrasonographic screening is thus extremely valuable for patients with chronic iron deficiency anemia presenting at around one year of age and relapsing after withdrawal of iron supplements, because no standard criteria for the acceptable age to perform small intestinal CE have been established.

In addition, elevated fecal calprotectin values and their utility as a diagnostic aid have been reported in colonic juvenile polyps [11]. Hestvik et al. reported that the median fecal calprotectin value was 75 μg/g (95% CI: 53–119) in healthy children aged one to four years [12], and Roca et al. recommended the cut-off value of 285.9 μg/g for healthy children aged one to four years [13]. Fecal calprotectin values measured at four points prior to polypectomy in the present case were 103, 134, 205, and 327 μg/g (in chronological order), demonstrating a similar level of elevation to that reported previously. However, no data related to fecal calprotectin in small intestinal juvenile polyps were found, and fecal calprotectin may also be useful as a diagnostic aid for isolated small intestinal juvenile polyps, although this was derived from the data of the present case alone. The present case is particularly valuable because the diagnosis of an isolated small intestinal juvenile polyp could be made based on the patient’s symptoms without surgical complications such as intussusception and is the first report of treatment by DBE.

Isolated small intestinal juvenile polyps may be treated non-surgically without intussusception developing with immediate diagnosis of the polyp using abdominal ultrasonographic screening and fecal calprotectin appropriately to make the differential diagnosis of protracted iron deficiency anemia.

## Figures and Tables

**Figure 1:**
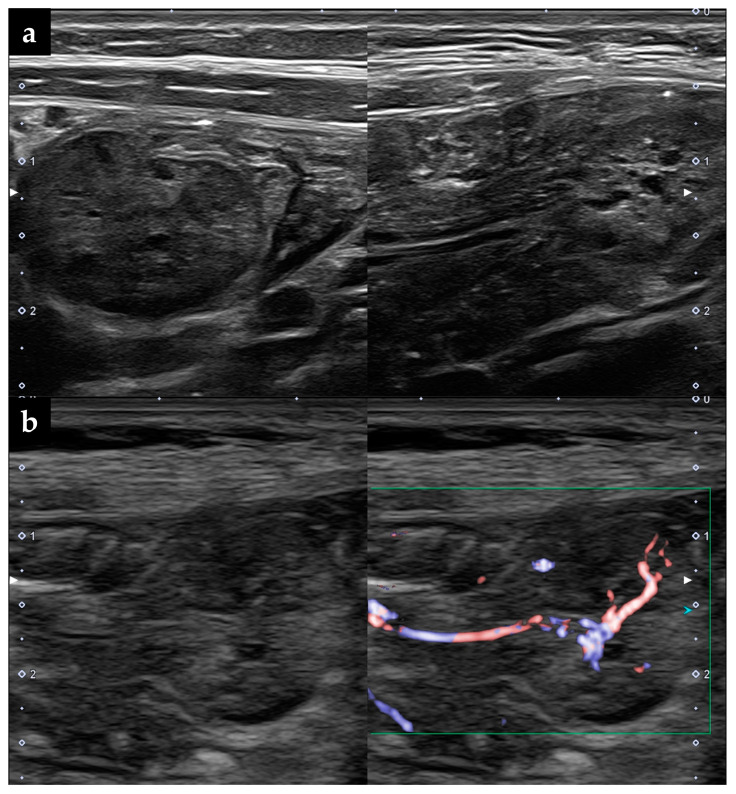
The ultrasonographic findings of the isolated small intestinal polyp. (**a**) Multiple anechoic cysts are identified within the parenchyma of the polyp. (**b**) The pedunculated polyp has a diameter of 15 mm and shows increased internal vascularity.

**Figure 2:**
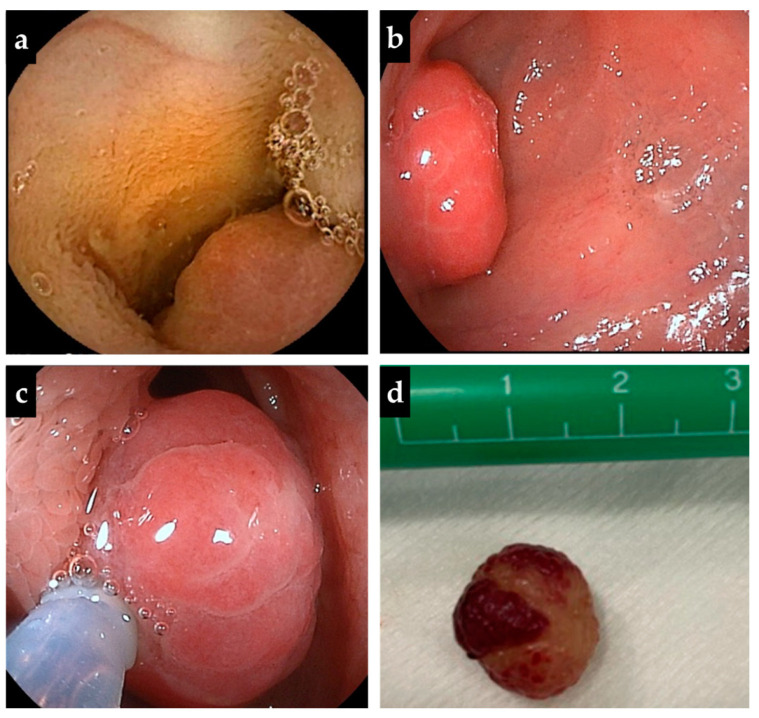
The findings of capsule endoscopy and double-balloon endoscopy. (**a**) Capsule endoscopy shows a polyp-like lesion at the upper jejunum, but no similar lesions are observed in other small intestinal segments. (**b**,**c**) A pedunculated polyp is found 75 cm anorectally from the pylorus and resected by cautery using a high-frequency snare. (**d**) The diameter of the resected polyp is 15 mm.

**Figure 3:**
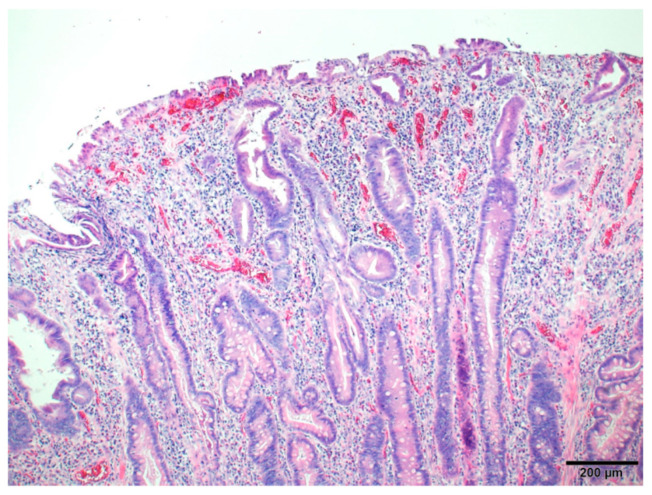
The histopathological findings of the resected polyp (hematoxylin and eosin staining). The histology shows mixed findings of dilated and serrated glandular ducts with partial granulation, consistent with a hamartomatous polyp.

## Data Availability

Not applicable.

## References

[1] Mandhan P. (2004). Juvenile colorectal polyps in children: Experience in Pakistan. Pediatr. Surg. Int..

[2] Yan J., Shen Q., Peng C., Pang W., Chen Y. (2022). Colocolic intussusception in children: A case series and review of the literature. Front. Surg..

[3] Van Lier M.G.F., Mathus-Vliegen E.M.H., Wagner A., van Leerdam M.E., Kuipers E.J. (2011). High cumulative risk of intussusception in patients with Peutz–Jeghers Syndrome: Time to update surveillance guidelines?. Am. J. Gastroenterol..

[4] Honda W., Ohmiya N., Hirooka Y., Nakamura M., Miyahara R., Ohno E., Kawashima H., AkihiroItoh, Watanabe O., Ando T. (2012). Enteroscopic and radiologic diagnoses, treatment, and prognoses of small-bowel tumors. Gastrointest. Endosc..

[5] Krasaelap A., Lerner D., Southern J., Noe J., Chugh A. (2020). Endoscopic removal of a single, painless, juvenile polyp in the small intestine causing anemia. J. Pediatr. Gastroenterol. Nutr..

[6] Kang S.I., Kang J., Kim M.J., Kim I.K., Lee J., Lee K.Y. (2014). Laparoscopic-assisted resection of jejunojejunal intussusception caused by a juvenile polyp in an adult. Case Rep. Surg..

[7] Ceccanti S., Frediani S., Manganaro F., Barbato M., Marcheggiano A., Cozzi D.A. (2012). Laparoscopic-assisted resection of juvenile polyp of the jejunum in a 3-year-old girl. J. Pediatr. Surg..

[8] Sah S.P., Agrawal C.S., Jha P.C., Rani S. (2002). Juvenile polyps in the small intestine presenting as jejunojejunal intussusception in a 10-year-old child: Report of a case. Surg. Today.

[9] Garcia Crespo J.M., Martin Pinto F., Dominguez Vallejo J. (1984). Intestinal polyp of infrequent localization: Presentation of 2 cases. An. Esp. Pediatr..

[10] Zimmermann H., Stauch G., Kamran D. (1981). Juvenile polyp in the small bowel (author’s transl). Z. Kinderchir..

[11] Das S.R., Karim A., RukonUzzaman M., Mazumder M.W., Alam R., Benzamin M., Marjan P., Sarker N., Akther H., Mondal M. (2022). Juvenile polyps in Bangladeshi children and their association with fecal calprotectin as a biomarker. Pediatr. Gastroenterol. Hepatol. Nutr..

[12] Hestvik E., Tumwine J.K., Tylleskar T., Grahnquist L., Ndeezi G., Kaddu-Mulindwa D.H., Aksnes L., Olafsdottir E. (2011). Faecal calprotectin concentrations in apparently healthy children aged 0-12 years in urban Kampala, Uganda: A community-based survey. BMC. Pediatr..

[13] Roca M., Rodriguez Varela A., Donat E., Cano F., Hervas D., Armisen A., Ana A., Maria J.V., Ander S., Ribes-Koninckx C. (2017). Fecal calprotectin and eosinophil-derived neurotoxin in healthy children between 0 and 12 years. J. Pediatr. Gastroenterol. Nutr..

